# Comparison of the efficacy and safety of selective internal radiotherapy and sorafenib alone or combined for hepatocellular carcinoma: a systematic review and Bayesian network meta-analysis

**DOI:** 10.1007/s10238-023-00997-3

**Published:** 2023-02-03

**Authors:** Hao Zeng, Chengyuan Zhou, Xiaojing Chen, Lanxin Hu, Ke Su, Lu Guo, Yunwei Han

**Affiliations:** 1 Department of Oncology, The Affiliated Hospital of Southwest Medical University, Luzhou, China; 2 Southwest Medical University, Luzhou, China; 3 Department of Ophthalmology, The Affiliated Hospital of Southwest Medical University, Luzhou, China

**Keywords:** Liver cancer, Non-surgical treatments, Yttrium-90, Transarterial radioembolization, Network meta-analysis

## Abstract

**Background:**

Selective internal radiation therapy (SIRT) is a developing technique and its efficacy and modality of application in hepatocellular carcinoma (HCC) are still controversial. This network meta-analysis aims to determine whether the efficacy and safety of SIRT alone and in combination are superior to that of sorafenib.

**Methods:**

Four databases (PubMed, Embase, Cochrane Library, and Web of Science) were searched before August 2022. Cochrane Randomized Trial Risk of Bias Assessment Tool and the Newcastle–Ottawa scale were used to assess the quality. The outcomes of interest included overall survival (OS), progression-free survival (PFS), and adverse events (AEs).

**Results:**

A total of 9 eligible trials involving 1954 patients were included, and SIRT ranked first among the three treatment modalities in terms of both OS (probability, 52.3%) and PFS (probability, 68.6%). The combination of SIRT and sorafenib did not improve OS or PFS in patients with HCC. Although the combination of SIRT and sorafenib did not raise the risk of grade 3 or higher AEs, it may have introduced more AEs than either alone.

**Conclusions:**

SIRT alone was found to be superior to sorafenib and the combination of the two in improving OS or PFS in patients with non-surgical HCC, especially in patients with combined portal vein tumor thrombus. The AEs induced by SIRT were different from those of sorafenib, but the overall toxicity was manageable, the combination of the two may cause an increase in the types of AEs that occur.

**Supplementary Information:**

The online version contains supplementary material available at 10.1007/s10238-023-00997-3.

## Introduction

Currently, effective treatments for HCC are limited. Surgical treatment, including hepatectomy and liver transplantation, represents the only curative treatment for HCC, which only applies to patients in the early stage [1, 2]. However, most patients were already locally advanced with vascular invasion or advanced stage with distant organ metastases at the first diagnosis and were not candidates for surgical treatment [3–5]. Sorafenib is an oral tyrosine kinase inhibitor with the ability to inhibit tumor cell proliferation and angiogenesis. It has been the first-line option for the group of patients with HCC since it received Food and Drug Administration (FDA) approval in 2008 [6, 7]. Although sorafenib improves overall survival (OS), the benefits are temporary and limited [5, 8].

When sorafenib has failed, targeted therapy like lenvatinib, regorafenib, and checkpoint inhibitors such as nivolumab monotherapy are systemic treatment options for advanced HCC patients [9]. Besides, local treatments such as radiotherapy are the other options that cannot be neglected. However, due to the poor tolerance of normal liver parenchyma to radiation, the application of external beam radiotherapy in HCC is limited for various reasons [10]. Selective internal radiation therapy (SIRT) involves the injection of yttrium-90 (Y-90) radioactive microspheres containing β-rays into the hepatic arteries under fluoroscopic guidance. Since healthy liver parenchyma is almost supplied by the portal vein, while tumor tissues are mainly supplied by the hepatic arteries, SIRT enables high-dose radiation therapy targeting with minimal radiation toxicity to normal liver parenchyma compared to external beam radiotherapy [11, 12].

Similar to sorafenib, SIRT is also available for unresectable HCC. Nevertheless, it is controversial whether SIRT prolongs survival and reduces the incidence of toxic side effects compared to sorafenib. According to the results of the Edeline, patients receiving SIRT had a longer median OS than those receiving sorafenib (18.3 vs. 6.5 months,* p* < 0.001) [13]. In contrast, in another study conducted by Chow et al. 2018, there were no significant differences in survivability between the SIRT and sorafenib groups (8.8 vs. 10.0 months, *p* = 0.36) [14]. At the same time, some researchers are focusing on whether the SIRT combination with sorafenib will achieve more significant efficacy. Ricke et al. compared the efficacy of SIRT in combination with sorafenib to sorafenib alone, while the study of Facciorusso et al. compared the combination group to SIRT alone [15, 16]. In addition, the safety of SIRT, a rapidly developing technology, alone and in combination with sorafenib, has also been a topic of interest for researchers [17].

In this systematic review and Bayesian network meta-analysis, by comparing the efficacy and safety of SIRT and sorafenib alone, and the combination of the two, we determined whether SIRT was as effective and safe as sorafenib alone. We aimed to evaluate the efficacy and safety of SIRT, sorafenib, and the combination in HCC, and to find the best strategy and population to benefit from the SIRT.

## Methods

This NMA is based on the PRISMA statement, which specifies the preferred reporting items for systematic reviews and meta-analysis. The protocol for this NMA is available in PROSPERO (CRD42022367650).

### Search strategy and selection criteria

Four databases (PubMed, Embase, Cochrane Library, and Web of Science) were searched before August 2022. The search strategy was provided as Supplementary material.

**Fig. 1:**
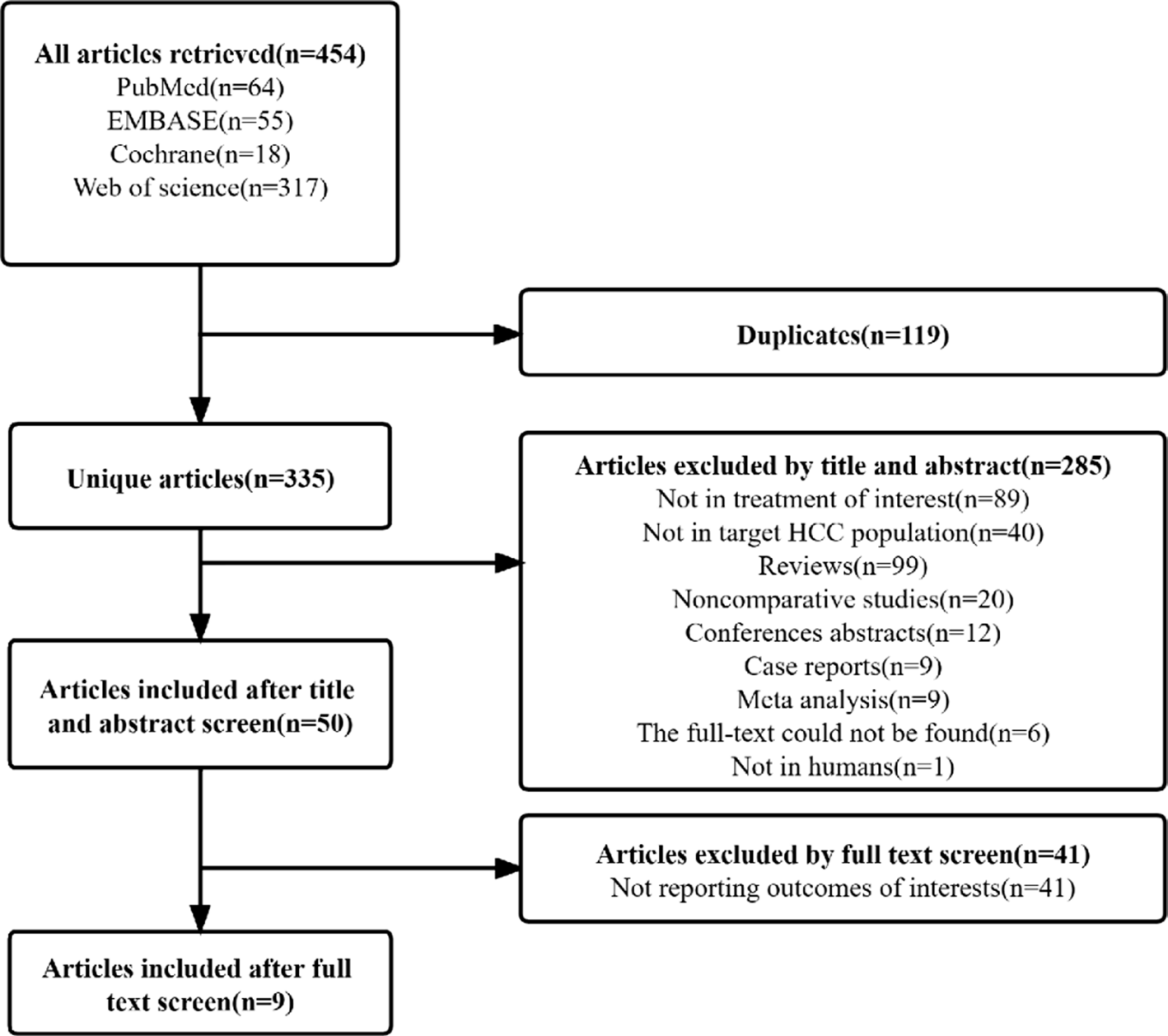
PRISMA flow diagram

All studies were screened for titles and abstracts by two independent reviewers. Literature screening is illustrated in Fig. 1. Clinical trials included in this NMA meet the following criteria: (1) participants should be at least 18 years old and have a histologically or radiographically confirmed diagnosis of HCC; (2) the included studies must compare at least two treatments for HCC, including SIRT, sorafenib, SIRT combine sorafenib; (3) the main outcomes assessed were OS and PFS; (4) observational studies and randomized controlled trials (RCTs) were included in the study design. Additionally, the articles in the reference list for the main studies were compiled. Study exclusion criteria are as follows: (1) conference abstracts, comment articles, case reports, non-comparative studies, or reviews; (2) languages other than English (3) no information on major outcomes; (4) duplicate articles.

The 2019 revision of version 2 of the Cochrane Randomized Trial Risk of Bias Assessment Tool (RoB2) was adopted for quality assessment in the current RCTs, the Newcastle–Ottawa scale was used to assess the quality of retrospective studies. The results of the evaluation are illustrated in Supplementary Table 1 and Supplementary Fig. 1.

### Data extraction and outcomes

We extract the following variables from each study: studies characteristics including authors, years, regions, interventions, and comparators of publication, patient baseline characteristics including number of patients, number of males, age, BCLC A/B/C, ECOG score standard, and Child–Pugh score intervention details. The hazard ratios (HRs) and 95% confidence intervals (CIs) of the primary outcomes (OS and PFS) provided by the studies were extracted. Secondary endpoint included specified grade ≥ 3 adverse events (Hypertension, Radiation hepatitis, Liver dysfunction, Ascites, Gastrointestinal bleeding, Abdominal pain, Nausea/vomiting, Diarrhea, Anorexia, Rash/desquamation, Weight loss, Fatigue, and Fever).

### Analyzed statistics

All data were analyzed in this study using software R (V.4.2.1) package gemtc (V.1.0-1) and Review Manager (5.4.1). The Bayesian NMA was performed to compare the interventions directly and indirectly, while pairwise meta-analyses were carried out for a direct comparison of SIRT versus sorafenib. Per-protocol (PP) populations were collected from three randomized controlled studies. The PP set is considered the preferred set for noninferiority investigation [18]. Therefore, hence PP was used for efficacy analysis, safety analysis, and subgroup analysis. The first step was to summarize the proof networks. A graph-splitting plot statistic was used to assess consistency, and a consistent ontology model was applied if *p* > 0.05. NMA determines whether to use a random or fixed effect model by model fitting. In the pairwise meta-analysis, random effects models were considered instead of fixed effect models since included studies were assumed to differ in clinical and confounding factors. The rank probabilities were used to evaluate and rank the regimes, and convergence was discussed in detail. The SUCRA (surface under the cumulative ranking curves) value and the area under the SUCRA curve are also calculated using R (V.4.2.1) to rank the efficacy of different interventions. SUCRA values ranged between 0 and 100%, with a higher value indicating higher effectiveness [19]. In addition, three subgroup analyses were conducted based on the pairwise meta-analysis (RCTs/non-RCTs, Asia–Pacific region/non-Asia–Pacific region, 100%PVTT/ < 100%PVTT).

## Results

### Identification and characteristics of eligible studies

Through a systematic search, 454 articles were discovered. After the removal of 119 duplicates, screening of the titles and abstracts of the 335 remaining articles was performed, and 285 articles were excluded for the following reasons: 89, were not in the treatment of interest, 40 articles were not in the target HCC population, 99 reviews, 4 conferences abstracts/case reports/meta-analysis, 1 animal studies, and 6 the full text could not be found. We conducted full-text reviews of 50 potentially eligible articles, and 41 articles were excluded for not reporting relevant outcomes. Finally, our NMA included 9 original articles, with a total of 1954 patients [13–16, 20–24]. The selection process for the study is illustrated in Fig. 1. A summary of study characteristics and study populations is provided in Supplementary Tables 2 and 3.

We evaluated 6 retrospective studies, three of which were of good quality and three of average quality. Three RCTs included 1 with low risk and 2 with some concerns. Details on the evaluation are provided in Supplementary Table 1 and Supplementary Fig. 1.

### Pairwise meta-analysis

**Fig. 2:**
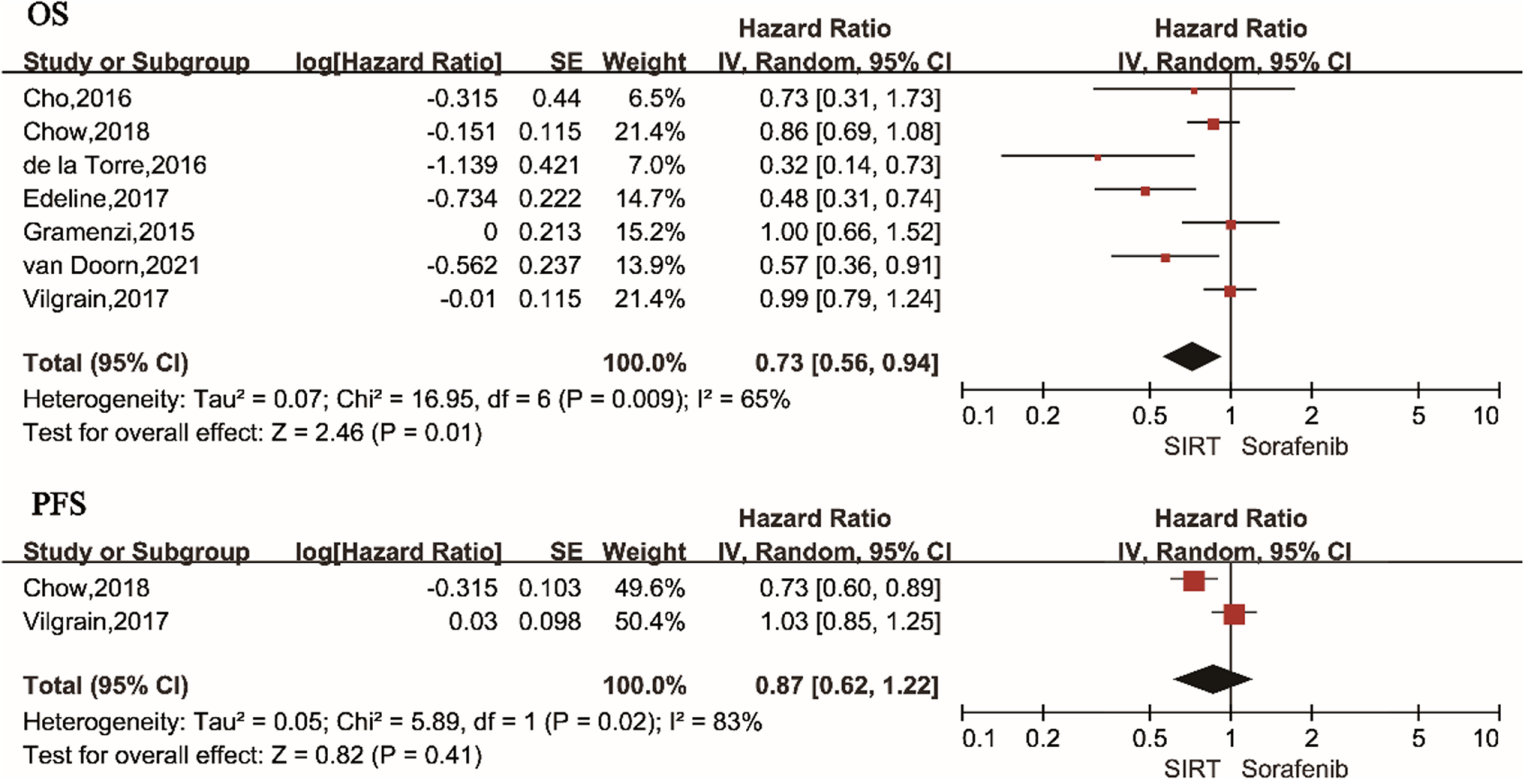
Forest plot of pairwise meta-analysis using a random effects model for overall survival (OS) and progression-free survival (PFS). The dotted line indicates the overall, pooled, estimate. The size of the shaded gray boxes indicates the relative weight of the study. CI: confidence interval

**Fig. 3:**
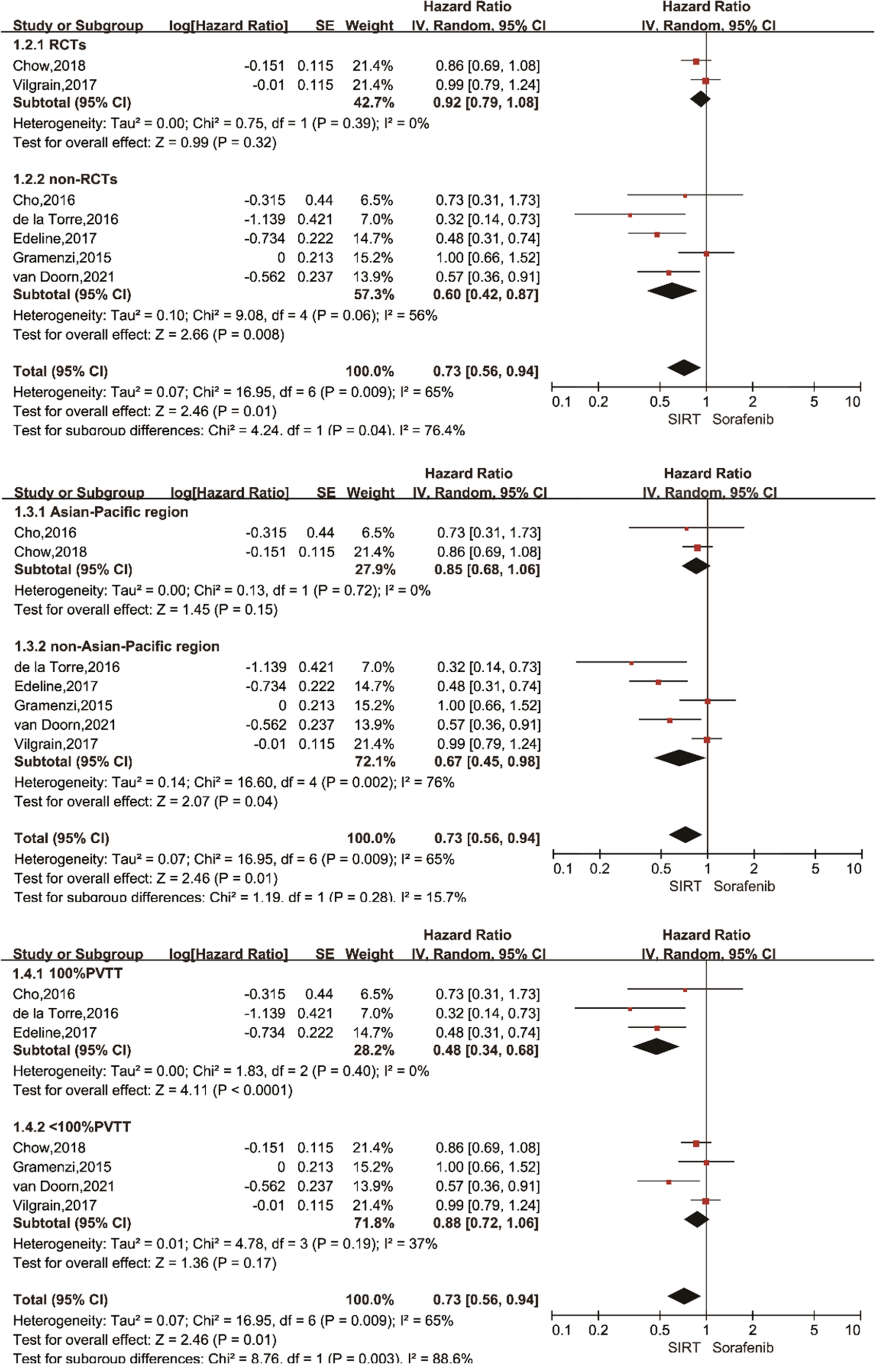
Subgroup analysis of randomized controlled trials (RCTs) (1.2). Overall survival (OS) for the sub-population from the Asian–Pacific region (1.3). OS for the sub-population with portal vein thrombus (PVTT) (1.4). CI: confidence interval

We included 7 [13, 14, 20–24] studies with SIRT and sorafenib interventions, and first conducted a systematic pairwise meta-analysis of these 7 studies. A record of OS is available for all 7 studies. There was a statistically significant difference in OS between SIRT and sorafenib groups in HCC (HR 0.73, 95% Cl 0.56–0.94; *p* = 0.01), and the studies were heterogeneous (*I*^2^ = 65%) (Fig. 2). We considered that heterogeneity may come from the design of the study, the area of the study, or whether all the subjects included had portal vein tumor thrombus. The subgroup analysis is carried out according to the above factors, and the following forest plot (Fig. 3) is obtained. Non-RCTs showed SIRT to be superior to sorafenib in OS (HR 0.60, 95% CI 0.42–0.87; *I*^2^ = 56%), while RCTs did not show a significant difference (HR 0.92, 95%CI 0.79–1.08; *I*^2^ = 0%); test for RCTs and non-RCTs differences: *I*^2^ = 76.4%. The Asian–Pacific region/non-Asian–Pacific region subgroup demonstrated no significant difference (Asian–Pacific region: HR 0.85, 95% CI 0.68–1.06; *I*^2^ = 0%; non-Asian–Pacific region: HR = 0.67, 95% CI 0.45–0.98; *I*^2^ = 65%); test for subgroup differences: *I*^2^ = 15.7%. There was a significant difference between the two groups when HR was pooled: 100%PVTT (HR 0.48, 95% CI 0.34–0.68) and < 100%PVTT (HR 0.88, 95% CI 0.72–1.06), heterogeneity between studies (100%PVTT: *I*^2^ = 0%, < 100% PVTT: *I*^2^ = 37%; Test for subgroup differences: *I*^2^ = 88.6%). Sensitivity analyses excluding the study from the analysis show that the main effect result remains robust. Only 2 [14, 23] of the 7 articles have a record of PFS. PFS of the SIRT group and sorafenib group was no significant difference (HR 0.87, 95% CI 0.62–1.22). (Fig. 2).

### Pooled results of Bayesian network meta-analysis

**Fig. 4:**
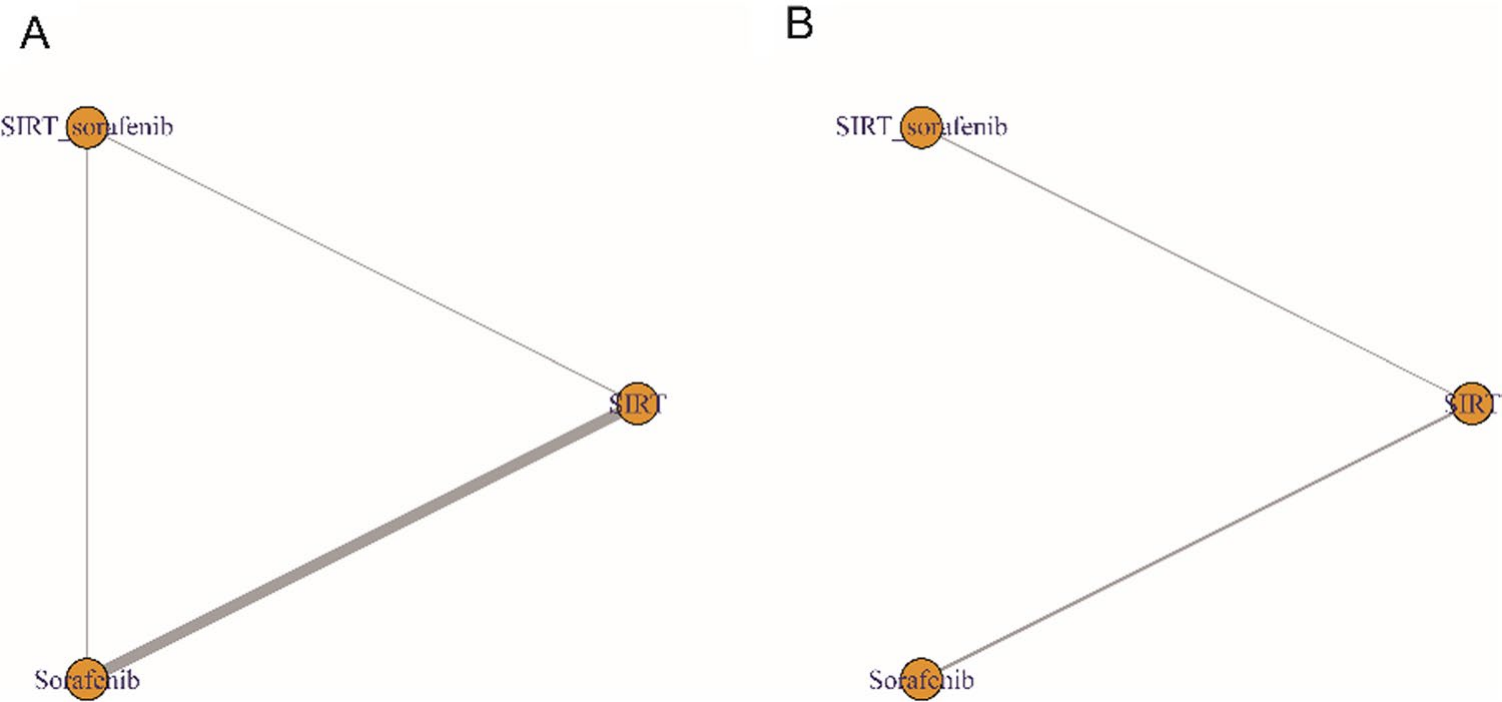
Network plot for overall survival (OS) (**A**) and progression-free survival (PFS) (**B**): The thickness of the connecting line corresponds to the number of trials between comparators. SIRT_Sorafenib: the combination of selective internal radiation therapy (SIRT) and Sorafenib

A total of 3 kinds of treatment (SIRT, sorafenib, SIRT plus sorafenib) were included in this study. Network traces of OS and PFS are shown in Fig. 4.

9 studies constituted a closed loop test implemented the inconsistency test (SIRT vs sorafenib: 7 studies; SIRT plus sorafenib vs SIRT: 1 study; and SIRT plus sorafenib vs sorafenib: 1 study), which showed that all the studies had no discrepancies in their OS and PFS, and there was no difference between direct and indirect evidence (all *p* > 0.05) (Supplementary Fig. 2). Therefore, a consistency model was applied. And no significant heterogeneity was observed between studies in Supplementary Fig. 7.

**Fig. 5:**
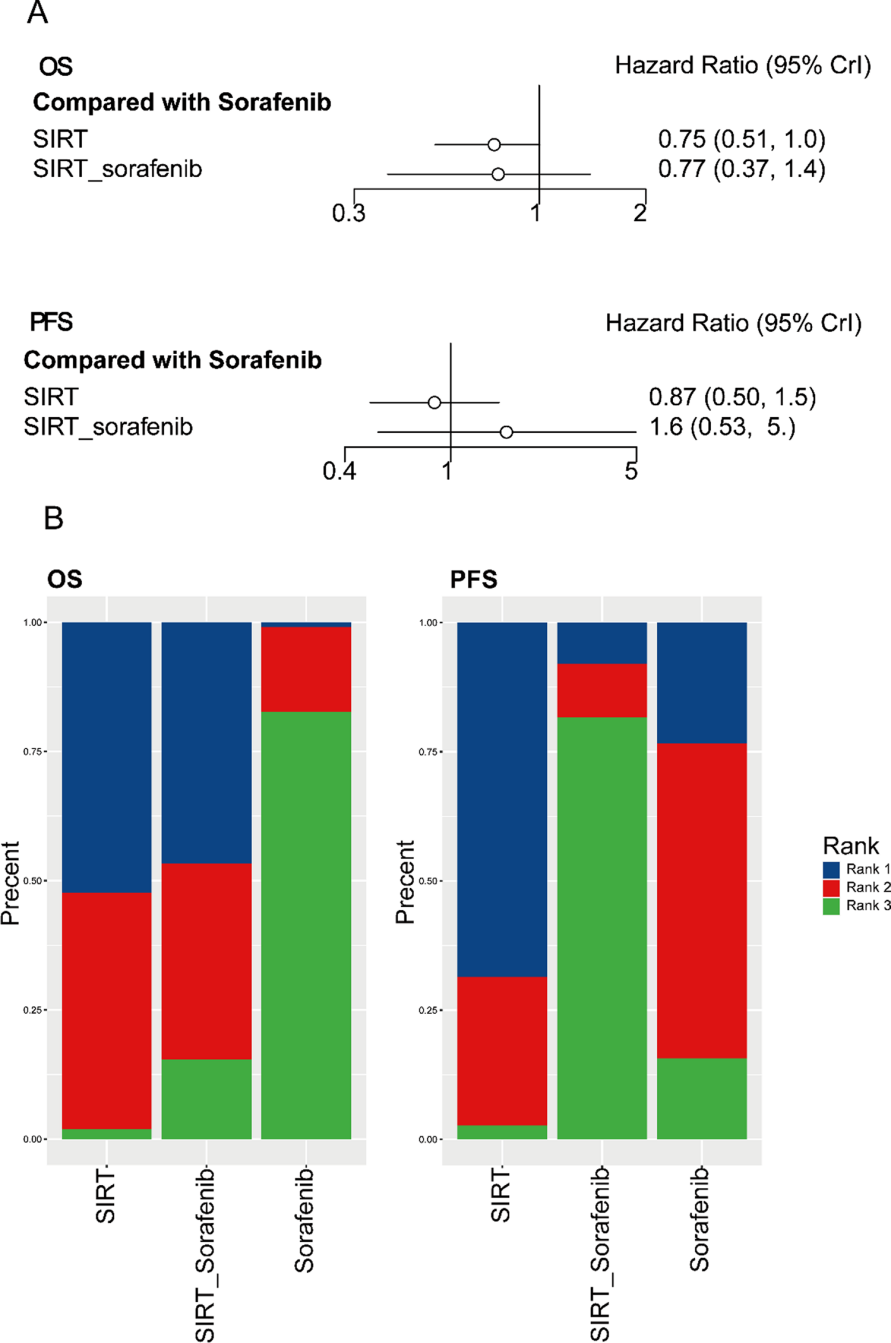
**A** Forest plot of Bayesian network meta-analysis using a random effects model for overall survival (OS) and progression-free survival (PFS). **B** Percent bar chart of overall survival (OS) and progression-free survival (PFS): Percent bar chart showing the likelihood of selective internal radiation therapy (SIRT), the combination of SIRT and Sorafenib (SIRT_Sorafenib), and Sorafenib being the most, second most, and least efficacious interventions in terms of OS in patients with hepatocellular carcinoma (left), and the likelihood of SIRT, Sorafenib, and SIRT_Sorafenib being the most, second most, and least efficacious interventions in terms of PFS in patients with hepatocellular carcinoma (right)

OS was reported in 9 studies, including 3 different interventions. Through model fitting, we find that the fitting effect of the random effect model (DIC: 16.94, ratio: 1.12, *I*^2^ = 20%) is better than that of the fixed effect model (DIC: 21.56, ratio: 2.17, *I*^2^ = 59%), so the random effects model was adopted. The comprehensive NMA was used to generate the pooled analysis and forest plots (Fig. 5). Compared with sorafenib, SIRT (HR 0.75, 95% CI: 0.51–1.0), and SIRT plus sorafenib (HR 0.77, 95% CI 0.37–1.4) were associated with better OS. The rank probabilities for all outcomes across all treatment comparisons are provided in Fig. 5. Detailed convergence-related discussions are performed by the node splitting method (Supplementary Figs. 3, 4).

PFS was reported in 3 studies [14, 15, 25] including 3 different interventions. We also find that the fitting effect of the random effect model (DIC: 6.29, ratio: 1.08, *I*^2^ = 38%) is better than that of the fixed effect model (DIC: 9.83, ratio: 2.60, *I*^2^ = 74%), an alternative model was adopted based on random effects. The pooled analyses and forest plots (Fig. 5) were generated using an extensive meta-analysis. Compared with sorafenib, there was a slight difference between the PFS of the SIRT (HR 0.87, 95% CI 0.50–1.5) and SIRT plus sorafenib (HR 1.6, 95% CI 0.53–5.). The sorting probability of all treatment comparison results is shown in Fig. 5. A detailed discussion of convergence is provided in Supplementary Figs. 5 and 6. In addition, SUCRA analyses indicated that OS and PFS were highest for SIRT (Supplementary Figs. 8 and 9).

### Adverse effects (AEs)

We found that sorafenib treatment led to a higher incidence rate of grade ≥ 3 AEs compared with SIRT, especially weight loss (SIRT vs sorafenib: 0.60% vs 1.83%; RR 0.328, 95% CI 0.067–1.615), diarrhea (SIRT vs sorafenib: 1.33% vs 5.95%; RR 0.224, 95% CI 0.080–0.630) and Rash/desquamation (SIRT vs sorafenib: 0.47% vs 3.15%; RR 0.148, 95% CI 0.035–0.626) than SIRT. And we compared the security of SIRT plus sorafenib and sorafenib, and the performance of sorafenib in terms of security is still not very satisfactory, especially in fatigue (SIRT plus sorafenib vs sorafenib: 1.92% vs 7.21%; RR 0.266, 95% CI 0.107–0.660), Rash/desquamation (SIRT plus sorafenib vs. sorafenib: 0.93% vs 3.15%; RR 0.294, 95% CI 0.069–1.242), Liver dysfunction (SIRT plus sorafenib vs sorafenib: 0.47% vs 6.51%; RR 0.071, 95% CI 0.0100–0.519) (Table 1).

**Table 1:** Network meta-analysis of grade ≥ 3 adverse events in SIRT alone, Sorafenib alone and SIRT combine Sorafenib

Toxicity	N	SIRT	SIRT_Sorafenib	Sorafenib	SIRT/Sorafenib			SIRT_Sorafenib/Sorafenib		
		No. Grade ≥ 3			RR	95% CI		RR	95% CI	
						Low	Up		Low	Up
Fever	5	3/422	1/261	4/535	0.951	0.214	4.225	0.512	0.058	4.562
Fatigue	5	28/520	5/261	48/666	0.747	0.476	1.174	0.266	0.107	0.660
Weight loss	3	2/332	NA	6/327	0.328	0.067	1.615	NA	NA	NA
Rash/desquamation	4	2/430	2/216	21/666	0.148	0.035	0.626	0.294	0.069	1.242
Anorexia	4	11/422	3/45	10/327	0.852	0.366	1.983	2.180	0.623	7.624
Diarrhea	3	4/300	6/216	30/504	0.224	0.080	0.630	0.467	0.197	1.105
Nausea/vomiting	6	8/552	4/261	6/697	1.684	0.588	4.824	1.780	0.506	6.258
Abdominal pain	5	12/552	3/45	16/489	0.664	0.317	1.390	2.038	0.617	6.729
Gastrointestinal bleeding	2	9/237	6/216	13/430	1.256	0.545	2.895	0.919	0.354	2.384
Ascites	6	27/554	12/261	26/784	1.470	0.867	2.490	1.386	0.710	2.708
Liver dysfunction	2	25/237	1/216	28/430	1.620	0.967	2.712	0.071	0.010	0.519
Radiation hepatitis	3	2/367	0/216	0/592	NA	NA	NA	NA	NA	NA
Hypertension	3	0/467	7/216	16/592	NA	NA	NA	1.161	0.484	2.785

*CI* Confidence interval, *N* Number of included studies, *RR* Risk ratio

## Discussion

The scenario of HCC is continuously evolving, and correspondingly, more effective and safe treatments are urgently needed [26]. According to this systematic review and network meta-analysis of HCC patients, we found that SIRT had advantages over Sorafenib for improving OS and PFS while the combination of the two was weaker than the SIRT alone group for improving OS and even worse than Sorafenib alone for PFS. Furthermore, SIRT also had an added benefit over Sorafenib in terms of safety.

To our knowledge, this is the first NMA comparing SIRT, Sorafenib, and the combination of the two. In a previous meta-analysis, Zou et al. included 6 original studies and found no statistical difference between SIRT and sorafenib alone in improving OS, and disease control rate (DCR) in HCC patients, but the SIRT group had a significantly higher objective response rate (ORR) than the sorafenib group, and the sorafenib group had a higher probability of grade ≥ 3 adverse reactions, which is similar to our findings [27]. Besides, Rognoni et al. have found that transarterial radioembolization (TARE), also referred to as SIRT, appears to be a reliable option for advanced HCC, especially in those with PVTT [28].

Generally, the formation of PVTT in HCC indicates the disease has progressed beyond the point of radical treatment. TACE(transarterial chemoembolization) is also contraindicated in the presence of PVTT [29]. SIRT is a microembolization procedure that minimizes changes in hepatic artery blood flow compared with other transarterial therapies [30]. Previous results from several retrospective studies of SIRT in patients with HCC combined with PVTT have shown that the efficacy and safety profile of SIRT was better than sorafenib [13, 21, 24]. This is consistent with the results of our subgroup analysis, in which SIRT prolonged OS more significantly in 100% of HCC patients with combined PVTT (HR 0.48, 95% CI 0.34–0.68). Currently, sorafenib tends to be the current primary choice for HCC patients with combined PVTT. Our results suggest that SIRT may be more beneficial in improving OS in this group of patients. Moreover, previous studies have indicated there were differences between East and West in approaches to treating hepatocellular carcinoma and the general lack of evidence related to the current use of SIRT in Asia [31]. Thus, we considered the differences due to the diverse study regions and performed subgroup analysis, concluding that the efficacy difference between Asia–Pacific and non-Asia–Pacific groups (*I*^2^ = 15.7%) was unapparent. Nevertheless, only 2 of the studies we included were from the Asia–Pacific region. To determine whether SIRT is reliable in Asian populations, more clinical studies in Asia are needed.

We included 2 clinical studies, which compared the efficacy of the combination group with that of sorafenib and SIRT, respectively. The findings of both studies showed that the combination of sorafenib and SIRT did not improve their efficacy, which was an unexpected result [15, 16]. As a local regional therapy, the killing effect of SIRT on local tumors leads to tumor ischemia, which in turn induces more production of VEGF and other hypoxia-inducing factors in the tumor microenvironment to counteract this alteration. At the same time, sorafenib is known to include the vascular endothelial growth factor receptor (VEGFR) among its targets, thus, the anti-angiogenic effect of sorafenib may provide theoretical support for alleviating the pro-angiogenic alterations brought about by SIRT [31, 32]. In fact, the efficacy of the combination group was disappointing. Further studies with larger sample sizes are needed on the combination of SIRT with sorafenib.

Safety was another important factor we considered. Consistent with previous studies, the incidence of grade ≥ 3 general adverse reactions were higher with sorafenib alone than with SIRT alone. This may be related to the nature of sorafenib as a multi-target kinase inhibitor, i.e., which refers to that sorafenib binds to the target in tumor tissue while also binding to the corresponding receptors in normal organs, resulting in multiple adverse effects throughout the body. Noticeably, although the probability of general adverse effects of SIRT was much lower, SIRT can also induce radioactive liver injury, i.e., damage to normal liver tissue due to the deposition of radioisotopes in blood vessels [33]. In previous findings, SIRT-induced radiological liver injury was considered acceptable compared to external exposure, whereas Ricke et al. have observed that the appearance of SIRT-induced radiological liver injury occurs at around 4 months, which is later than expected, as this tends to occur at an early stage after receiving radiation [34]. In the SIRT and sorafenib combination group, Facciorusso et al. concluded that treatment toxicity and liver failure rates in this group were similar to those in the SIRT alone group and that the combination of sorafenib and SIRT was feasible and tolerated [15]. However, Ricke et al. concluded that because the toxicity of the two did not overlap, the odds of grade 3–4 adverse reactions were higher in the combination than in the single-use group, without increasing the incidence of grade 5 adverse reactions [16]. The management, treatment outcome, etc. of HCC is continuously and rapidly changing, according to an updated survey, a continuous monitoring is required to determine how changes in the clinical history of HCC will affect treatment [26]. Therefore, the usage of SIRT, especially in combination with sorafenib, should pay attention to the hepatic function status of the patients, especially to the chronic damage caused by SIRT on the patient's liver function.

To sum up, SIRT may no longer be considered an experimental treatment with improved techniques, standardized procedures, and the experience that has been accumulated in numerous centers. The combination and the sequence of use among SIRT and other treatments should be given more attention in the application of SIRT. At the same time, due to the chronic liver function damage caused by SIRT that cannot be ignored, more careful consideration should be given to patient selection, and particular attention should be paid to the liver function status of the patients to ensure the efficacy of SIRT while protecting their normal liver function as much as possible.

There still are some limitations in our study. Firstly, only 3 of the 9 studies we included were RCTs, and the rest was retrospective studies, limited by sample size and patient selection, which inevitably led to heterogeneity in this study. Secondly, because of the limited number of original studies in the combination group of SIRT and sorafenib, we included only 2 studies, which may lead to bias in our observation of the true efficacy of the combination of SIRT and sorafenib. Thirdly, since van Doorn, Gramenzi, and Facciorusso did not publish the complete data of their study, the data was extracted from the figures we present in the text and therefore some potential bias may arise. Given these limitations, additional randomized controlled clinical studies are needed to validate our results.

## Conclusions

In this Bayesian network meta-analysis, SIRT alone appeared to be the best option to improve OS and PFS in HCC patients compared to sorafenib and SIRT in combination with sorafenib, especially in patients with combined PVTT. We also found that the safety of SIRT was overall superior to that of sorafenib and that when they were combined, more different toxic effects might be introduced, although the risk of serious adverse reactions was not elevated. The findings could complement the current state of research on the use of SIRT and enhance the design of future clinical trials of SIRT in HCC.

### Supplementary Information

Below is the link to the electronic supplementary material.

Supplementary file1 (DOCX 1921 KB)
